# The Impact of Prenatal Exposure to Bisphenol A on Male Reproductive Function

**DOI:** 10.3389/fendo.2020.00320

**Published:** 2020-05-29

**Authors:** Roger J. Hart

**Affiliations:** ^1^Division of Obstetrics and Gynaecology, University of Western Australia, Perth, WA, Australia; ^2^Fertility Specialists of Western Australia, Bethesda Hospital, Claremont, WA, Australia

**Keywords:** BPA, sperm count, testosterone, male reproduction, raine study, endocr disrupting chemicals, early life exposures, *in-utero*

## Abstract

Bisphenol A (BPA) is a recognized xenoestrogen, in that it possesses oestrogenic and anti-androgenic properties. These endocrine-disrupting effects of BPA at the estrogen receptor (ER) occur despite the very low affinity of BPA for the ERβ, which is 10,000 times lower than that of 17-β estradiol, and despite the European regulatory authorities stating that BPA is safe, at usual exposure concentrations, the use of BPA in baby drink bottles was banned in 2011. There exists conflicting evidence from human epidemiological studies as to its influence on adult male reproductive function, although animal data is more convincing. This mini-review will report on the limited epidemiological data from human studies relating early life exposure to BPA on adult male reproductive function. A long term follow-up study from Western Australia using a birth cohort, the Raine Study, demonstrated no adverse associations of antenatal exposure to BPA, and potentially a positive association with antenatal BPA exposure with sperm concentration and motility at 20 years of age, although recent scientific reports suggest traditional measures of BPA exposure may underestimate exposure levels, which makes data interpretation potentially flawed.

## Introduction

Bisphenol A (BPA) is a widely used chemical which is ubiquitous within the environment, being present within plastics and epoxy resin. In the United States the Centre for Disease Control and Prevention reported that more than 90% of individuals, in the early years of the twenty-first century, had measurable concentrations of BPA present within their body (1). The production of BPA has increased substantially over the last 15 years and the projection for 2020 is 9,600 kilo tons http://www.digitaljournal.com/pr/2009287 (2). Exposure to BPA can be through the diet, drinking, inhalation or dermal contact, although inhalation exposure appears to be negligible in comparison to the dietary route (3). Furthermore, measurable levels of BPA have been detected in breast milk, amniotic fluid, and cord blood. Furthermore, the fetus is at risk of BPA exposure as it freely crosses the placenta. In the circulation BPA is present in the free form at about 8% of the total BPA in the blood (4). Subsequent to eating, after gastric absorption, peak serum BPA concentrations are reached within 90 min (5), and BPA is rapidly eliminated, after gut absorption (6), dermal and sub-lingual absorption have different pharmacokinetics. After undergoing rapid conjugation, forming inactive glucuronides and sulfates by the liver of the mother and fetus, and by the placenta, BPA is excreted in the urine (7), although some work suggests that the glucuronide metabolite may be active (8). Consequently, it is theoretically possible for the fetus to be exposed to a greater concentration of BPA than the mother, as the placenta can de-conjugate BPA by placental sulphatase and beta- glucuronidase enzymes (9), and furthermore, the immaturity of fetal liver would make the BPA conjugation poorly effective in the fetus (9). However, the significance of this placental metabolism is believed to be low (7), although studies suggest almost universal exposure of pregnant women to BPA, and a substantial variation in its metabolic clearance, which will lead to substantial variability of fetal exposure (10).

Despite reassurances of the safety of BPA by the European Food Safety Authority (EFSA) as recently as 2015, the EFSA reduced the tolerable daily intake of BPA from 50 μg/kg body weight per day (bw/day) to 4 μg/kg bw/day, and stated that the average daily exposure was below this “safe” level (11). With estimated BPA dietary intake in infants and toddlers (up to 0.875 μg/kg bw/day), with reproductive aged women having dietary exposures comparable to men of the same age (up to 0.388 μg/kg bw/day), and adolescent exposure of upto 1.449 μg/kg bw/day, in 2011 the European Union banned the use of BPA within baby bottles. Interestingly, due to the lipophilic properties of BPA, BPA could concentrate in the breast milk, and levels of infant exposure to BPA decrease with the introduction of solid foods (11).

Due to its prevalence within the environment, and its known endocrine disrupting effects, it has been suggested that BPA may have a negative impact on male fertility acting as a xenoestrogen. Unconjugated BPA binds as a weak agonist to estrogen receptors α and β (12, 13), as well as the androgen receptor (14). Hence, it may be expected to potentially impact the reproductive development of the male, particularly if exposure was to occur during a vulnerable period of development of the male fetus during pregnancy. It has been demonstrated in rodent models that a “masculinization programming window” exists in pregnancy, and would be expected to correlate with 8–14 weeks gestation in humans (15). Features of lack of male androgenisation are a shorter anogenital distance, impairment of sperm production, hypospadias and cryptorchidism (15), which have been grouped together as part of a “testicular dysgenesis syndrome” (TDS) (16, 17). Consequently, it is during this period of time that the male fetus would, theoretically, be at greatest vulnerability to chemicals that either interfere with the secretion, transport, action, metabolism, and excretion of testosterone; the hormone primarily responsible for fetal masculinisation. This is particularly of relevance as BPA freely diffuses across the placenta (7), and the placenta's ability to conjugate, and hence potentially de-activate BPA is limited. Hence, BPA at maternal serum concentrations may freely pass to the fetus across the placenta, leading to near-equivalent levels in fetal and maternal blood (7), therefore measuring maternal circulating concentrations is a reasonable proxy for fetal exposure.

It has been assumed by many experts that sperm counts may have been diminishing over the last 30 years, although this is hotly debated (18, 19), however it is not disputed that the incidence of undescended testis, hypospadias and testicular cancer is increasing in some countries (20–23). The TDS hypothesis proposes that, as a result of abnormal testicular development, a secondary abnormality in Leydig and/or Sertoli cells results during male sexual differentiation, leads to reproductive disorder in later life (24, 25), again, this assertion has been disputed (26). However, with the increasing prevalence of oestrogenic endocrine disrupting chemicals within the environment it is plausible, but unproven, that human fetal Sertoli cell proliferation may be altered by an excessive oestrogenic environment in early life. Consequently, researchers have attempted to study potential associations of early life exposures to oestrogens (27), and endocrine disrupting chemicals (28, 29), with the incidence of cryptorchidism (30), anogenital distance (a reliable marker of prenatal androgenisation) (31), pubertal timing (32), sperm counts (27), and adult markers of testicular function (27). This mini-review will review the epidemiological studies of prenatal BPA exposure on human male reproductive function.

## Background Animal Studies of Exposure to BPA

Data from animal studies provide potential mechanistic insights to the human data and are briefly reviewed for context. Animal studies suggest that exposing mice early in the neonatal period to BPA, at concentrations that humans encounter daily, may reduce sperm number, motility, and maturation, without influencing testicular histology (33). Perinatal BPA administration to female rats has been reported to reduce the fertility of the mature male offspring (34). Furthermore, negative influences on plasma testosterone and estradiol concentrations have been reported subsequently, after maturity, when pre-pubertal rats were exposed to low doses of BPA, inducing some degree of androgen deficiency features in adulthood (35, 36).

**Figure 1:**
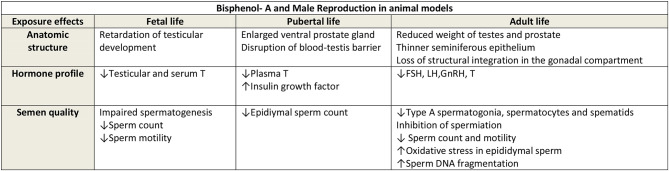
Reproduced with permission Cariati et al. (44). FSH, Foliular stimulating hormone; LH, Luteinising hormone; GnRH, Gonadotrophin releasing hormone; T, Testosterone.

Male mice exposed *in utero* to BPA have been demonstrated to have reductions in concentrations of serum and intra-testicular testosterone (37), impairments of testicular development (37) and spermatogenesis (37), with reduced sperm counts (38). Indeed, studies suggest that BPA may be a testicular toxicant in animal models (39, 40). Furthermore, adverse effects of BPA exposure on rodents' developing testis and prostate stem cells have been also reported (41, 42). Other animal studies suggest that BPA may exert it effects through central influences from *in-utero* maternal BPA exposure causing alterations in gonadotrophin releasing hormone and kisspeptin secretion, and consequently influence anterior pituitary function (43). From Figure 1 (44), it can be seen that the influence of BPA exposure at different stages of development in the animal model appears to produce similar effects on reproductive function in adulthood. Due to the concerns of the potential health effects of BPA on human health analogs to BPA have been introduced into commercial production. However, this approach may not be entirely beneficial, as one study that administered BPA, and its analogs bisphenol B, bisphenol F, and bisphenol S, at various low concentrations to pregnant rats, demonstrated in the male offspring a decrease in sperm production, testosterone secretion, and histological changes in the reproductive tissues with these analogs (45).

## Human Studies of Prenatal Exposure to BPA

Due to the difficulty of completing human studies, there are understandably less studies that have addressed human prenatal exposure to BPA on subsequent male reproductive development. This is in part due to the duration of follow-up required to study potential exposure effects, the potential multiple confounders inherent in any human exposure study, and consequently the cost of such long-term studies. As the measured anogenital distance (AGD) is now a recognized marker of prenatal androgenisation (46), with a longer AGD being a marker of greater prenatal androgen exposure, this offers a potential reference point to assess prenatal androgenisation. Researchers from Shanghai measured the AGD of male infants, and related this distance to the maternal urine BPA concentration at 12–16 weeks of gestation (47). This early stage of pregnancy is considered a critical time for prenatal androgenisation, as described the masculinization programming window (48), where perturbations in the androgenic environment, may have long term consequences. This study demonstrated that those boys, whose mothers had detectable levels BPA in their urine, at 12–16 weeks of gestation, were more likely to have shorter AGD at birth, than boys with undetectable levels of maternal BPA (47). These findings were consistent when measured again at both 6 and 12 months of age, and was irrespective of ascertainment of AGD by measuring from the anus to the base of the penis, or the scrotum (47). A further study was performed using cord blood measurements of BPA in relation to the AGD among 72 boys, which demonstrated an inverse relationship between cord blood BPA concentrations and male newborn ano-scrotal distance (49).

With respect to pubertal timing, a recent study demonstrated an association of greater prenatal exposure to BPA, assessed by maternal urine measurement, with later puberty in girls and earlier puberty in boys (50). Nevertheless, when data were adjusted for overweight/obesity status, all associations for boys were attenuated, suggesting a contribution of body fat in mediating the associations (50). An earlier study, possibly the first reported study, of BPA exposure as assessed by a 3rd trimester urine sample relating exposure to pubertal timing, did not demonstrate any association with hormone levels or pubertal staging in adolescence (51). However, this may relate to the sampling timing in this study not being performed at a vulnerable time in pregnancy.

With respect to deriving associations of *in-utero* exposures to BPA with adult reproductive assessment only one study has been undertaken (28). This study, led by the author of this mini-review, studied early life influences on adult testicular function. This was a birth cohort study where men from the birth cohort, who had been followed very closely through childhood, were recruited at 20 years of age to undergo a thorough testicular assessment (serum sex steroids and gonadotrophins were measured, semen assessment undertaken, and a testicular ultrasound examination performed). The mothers of 149 of the men had blood drawn at 18 and 34 weeks of gestation in 1990–1991, and their paired samples were mixed to provide an “average” of antenatal exposure. The total BPA concentrations in the maternal samples were measured by mass spectrometry and correlated with their sons' adult testicular function. There was a substantial range in serum concentrations measured in maternal serum, with the 10, 25, and 95th percentile concentrations recorded as ≤ 0.005, 0.08, and 2.15 μg/l, respectively, reflecting a large variation in exposures. The result of the analysis after adjustment for time since last ejaculation, maternal smoking, sexual abstinence, and presence of a varicocele, demonstrated that maternal BPA exposure was positively associated with their sons' sperm concentration and motility in adulthood. In addition, no associations of maternal serum BPA concentrations were detected with their sons' testicular volume or hormonal measures of male reproductive function in adulthood; serum testosterone, LH, FSH, or inhibin B concentrations (28). The positive association of maternal BPA exposure with sperm concentration and motility may be chance findings (Table 1), in view of the lack of other associations being identified. However, the association may be real, but the study is limited by numbers of participants (maternal BPA measures were available for 284 men, however only 149 of them underwent testicular assessment at 20 years of age). It is important to state that contemporary BPA exposure was not measured, which may have influenced the results, as recent xenoestrogen exposure has the potential to influence the testicular assessment. Furthermore, any potential associations may be lost by the long duration of follow-up, due to the multiple exposures and life events that will have occurred over 20 years. Within our study the median total serum BPA concentration within the maternal blood was 0.25 μg/l, which is similar to reported by the EFSA (11), and other authors (52, 53). However, it must be stated that the assessment of BPA exposure was via serum sampling, whereas the standard method of assessment is urine, hence the serum concentration documented may not reflect a more sustained exposure as recorded in urine measurement. As the concern with serum measures is that urine provides significantly less variability than serum for a compound with a relatively short half-life, although even urinary total BPA concentrations vary across different times in pregnancy (54), and individuals have a diurnal variation, with the exposure levels generally being lower in the morning than the evenings (55). Consequently variability of the concentrations recorded understandably reduces the power of any statistical analysis. Furthermore, as recently proposed, if the method of analysis of BPA concentrations was flawed, then the exposure levels may have been greater than reported, and subtle associations may have been missed (56), although the recognized measurement of serum BPA is well-established and reliable, as BPA contamination can be controlled during sample collection and inadvertent hydrolysis of BPA conjugates can be avoided during sample handling (57, 58).

**Table 1:** Correlation between adult testicular volume and semen parameters with BPA exposure.

**Ranked phthalates**		**Testis volume (mls)**	**Semen sample parameter**					
			**Volume**	**Sperm output**	**Concentration**	**SCSA**	**Normal morphology**	**Motility**
			**(mls)**	**(million)**	**(million/ml)**	**(%)**	**morphology**	**(a + b grade)**
BPA	Correlation	−0.05	−0.05	0.13	**0.18**	0.05	0.00	**0.18**
	*P*-value	NS	NS	NS	**0.037**	NS	NS	**0.036**

*All correlations were adjusted for abstinence period, presence of a varicocele and maternal smoking and in addition, testicular volume was also adjusted for adult height (z-scores). NS, not statistically significant; BPA, bisphenol A; SCSA, sperm chromatin structural assay*.

*Values in bold purely highlighting the statistically significant results*.

## Conclusions

The focus of this mini-review has been to determine if there is any association between prenatal BPA exposure and human male reproductive function. There have been many cross-sectional studies looking at linking assessment of reproductive function with current BPA exposure, such as timing of puberty and sperm counts, however the purpose of this review was to determine if the exposures to BPA at a critical stage of development, the “masculinization programming window” may lead to a permanent perturbation in the hypothalamic-pituitary-gonadal axis. Furthermore, from animal studies it may be suspected that BPA exposure may also have a permanent gonadotoxic effect. The benefit of animal studies are numerous, in that they are comparatively cheap, have the ability to control for multiple confounders and exposures within an environment, and due to their short gestation, and pubertal maturation period, provide an ability to review a life-span in a relatively short period of time. However, a major problem with animal studies of endocrine disrupting chemicals is that these chemicals are known to have potential different effects at different concentrations leading to difficulty in extrapolating animal effects to the human situation. Furthermore, whilst it appears form the animal studies that BPA has an endocrine disrupting influence when administered in the prenatal, and perinatal period, it is important to determine whether human exposures are at, above or below, safe levels of exposure in the perinatal period. Controversially, the EFSA stated in 2015 that current levels of exposure are below the tolerable daily intake (<4 μg/kg bw/day) and as such current BPA exposure does not pose a threat to the fetus (11). However, work performed by independent researchers cast some doubt on these claims, and raise concerns that very low doses of exposure may pose a risk during development (59, 60). Furthermore, there is evidence to suggest that the previous methods used to measure BPA exposure, using enzyme de-conjugation, may substantially underestimate human exposure, and hence fail to detect any subtle associations (56). The explanation for this is that an assay that reduces the variance in BPA concentrations, underestimates the exposures of those most highly exposed, tends to lead to an increase in the likelihood of false negative findings. Furthermore, it is proposed that the current safe levels are flawed, as evidence suggests that low-dose BPA exposure induces marked adverse effects below the considered safe levels (40). Indeed, the greatest number of effects were observed, in one study, at doses substantially lower than the current “safe” dose of BPA for humans (59). As this CLARITY study found that there were evidence of detrimental effects detected at doses of 2.5 μg/kg per day (59). With respect to human serum levels of free BPA, the serum concentrations have been reported to be below the limits of detection (<0.2 μg/L) in several cohorts (11, 52), which has led to doubts around the potential for environmental BPA exposure to exert endocrine disrupting effects (52).

The limited human data presented suggest that prenatal exposure to BPA may have a potential negative association with early life anogenital distance for boys, but the evidence for an influence on pubertal timing is less clear. Furthermore, it is unclear whether prenatal exposure to BPA *in-utero* has an influence on later life mature male reproductive health, with the data suggesting a potential positive association with sperm concentration and motility at 20 years of age. Certainly there is a need for further long-term studies of early life exposure to endocrine disrupting chemicals, such as BPA, to assist individuals and regulatory authorities in their decision making for the use of chemicals in the environment.

## Author Contributions

RH conceived the mini-review and wrote the manuscript.

## Conflict of Interest

The author has equity interests in Western IVF.
